# Negative and Positive Body‐Related Emotions Derived From Voice Recordings During a Mirror Task in Anorexia and Bulimia Nervosa: A Natural Language Processing Approach Using RoBERTa

**DOI:** 10.1111/eat.70007

**Published:** 2025-11-26

**Authors:** Linda Marie Sadowski, Christopher Lalk, Vanessa Hofschröer, Fanny Alexandra Dietel, Julia Tanck, Julian A. Rubel, Andrea S. Hartmann, Silja Vocks

**Affiliations:** ^1^ Institute of Psychology, Department of Clinical Psychology and Psychotherapy Osnabrück University Osnabrück Germany; ^2^ Institute of Psychology, Department of Psychotherapy Research and Clinical Psychology Osnabrück University Osnabrück Germany; ^3^ Department of Psychology, Workgroup of Clinical Psychology and Psychotherapy of Childhood and Adolescence University of Konstanz Konstanz Germany

**Keywords:** body image, disgust, eating disorder symptoms, emotion analysis, machine learning, mirror exposure, natural language processing, negative emotions, positive emotions, RoBERTa

## Abstract

**Objective:**

Body dissatisfaction has been linked to negative and positive emotions. The validity of self‐report methods to assess emotions in individuals with eating disorders is limited, prompting a shift towards methods like natural language processing to analyze speech content. Using artificial intelligence, this study aimed to identify specific body‐related emotions elicited by looking at oneself in the mirror in individuals with eating disorders.

**Method:**

Women with anorexia nervosa (*n* = 24), women with bulimia nervosa (*n* = 36), and healthy women (*n* = 72) completed a three‐minute mirror exposure task, verbally expressing their body‐related emotions while viewing themselves in a three‐winged mirror wearing underwear. *N* = 132 audio recordings were transcribed and analyzed using the *GermanEmotions* model (based on *RoBERTa*) to identify 28 emotions. Univariate ANOVAs and post hoc tests were conducted to identify group differences in expressed emotions. Prediction of symptom severity was analyzed across groups.

**Results:**

Compared to healthy women, those with anorexia and bulimia nervosa expressed anger, disappointment, disgust, embarrassment, fear, grief, nervousness, remorse, and sadness significantly more frequently (all *d*s = |0.53|–|1.11|), and admiration, approval, joy, love, optimism, and pride significantly less frequently (all *d*s = |0.64|–|1.12|). Disgust predicted higher eating disorder symptom severity across all groups (*p* < 0.001).

**Discussion:**

A distinct range of body‐related emotions differentiates women with anorexia and bulimia nervosa from healthy women. Elicited negative emotions, especially higher disgust, and diminished positive emotions suggest that body‐related interventions could benefit from fostering positive emotions instead of merely reducing negative emotions.

## Summary

- This study analyzed voice recordings collected during a standardized mirror task in women with anorexia or bulimia nervosa, and healthy women, employing artificial intelligence to extract individual negative and positive emotions.
- Utilizing a state‐of‐the‐art natural language processing approach, we identified specific heightened negative emotions and diminished positive emotions in women with anorexia or bulimia nervosa versus healthy women.
- Disgust appeared to be the most prominent emotion across all groups.

## 1 Introduction

Dissatisfaction with one's body is widespread in the general female population (Quittkat et al. 2019) and constitutes a core symptom of eating disorders (ED; Tylka 2004). Relatedly, cognitive‐behavioral theories on the etiology and maintenance of ED include excessive concerns about one's body size and shape (Fairburn et al. 2003; Williamson et al. 2004). These theories further propose that body‐related stimuli (e.g., seeing one's reflection in the mirror) activate negative body‐related schema and associated cognitive biases (Williamson et al. 2004), in turn fueling negative emotions (e.g., anger, disgust, anxiety) and thereby potentially exacerbating ED symptoms as individuals seek to relieve emotional distress, for instance through body checking or restrictive eating (Cordes et al. 2015; Williamson et al. 2004). Overall, these theoretical assumptions emphasize the centrality of body‐related emotions in ED maintenance, underlining the need to understand their phenomenology to inform both the theory and treatment of ED.

Previous research has consistently shown that negative body‐related emotions are generally associated with disordered eating behaviors, such as restrictive eating, excessive exercising, or avoidance of body‐focused situations in women with anorexia nervosa (AN; e.g., Espeset et al. 2012). Furthermore, studies investigating the role of specific emotions have reported associations between eating disorder pathology and body‐related guilt, shame, envy, and embarrassment in undergraduate women (Solomon‐Krakus et al. 2022), and disgust in women with AN (Kot et al. 2021). However, as has been extensively demonstrated (Blome and Augustin 2015), retrospective assessments are prone to biases such as recall bias, with reflection on past emotional experiences typically leading to an under‐ or overestimation of emotional intensity or valence (Colombo et al. 2020). Moreover, broad retrospective assessments may lack ecological validity, as they do not involve the presence of an eliciting stimulus. To counteract these undesired effects, some studies have sought to induce body‐related emotions within pertinent, disorder‐relevant situations. For instance, studies investigating body‐related emotions during mirror exposure found that individuals with AN (Vocks et al. 2007), bulimia nervosa (BN) and eating disorders not otherwise specified (EDNOS; Trentowska et al. 2013; Vocks et al. 2007) experienced more negative emotions than healthy women. Relatedly, studies investigating the occurrence of positive emotions during mirror exposure or when viewing pictures of oneself in a bikini (Vocks et al. 2010) found that women with BN experienced fewer positive emotions than healthy women. However, a further limitation across these studies is their use of limited, predefined categories to assess emotional state. Specifically, to date, there are only a few self‐report questionnaires covering a variety of emotions, with some related to the body (e.g., the Body and Appearance Self‐Conscious Emotions Scale assessing guilt, shame, pride; Castonguay et al. 2014). Beyond these, most studies employed preset rating scales for emotions based on previous research (e.g., sadness, tension, fear, insecurity, and disgust; Vocks et al. 2007; or sadness, disgust, anxiety, distress, and insecurity; Trentowska et al. 2013) or clinical experience (e.g., stress and anger; Vocks et al. 2007). Consequently, given these methodological limitations, the aforementioned studies fail to capture the full spectrum of body‐related emotional experiences, resulting in an incomplete understanding of predominant emotional states.

One way to address such limitations is the direct analysis of spoken language, which offers the intriguing possibility to assess momentary emotional processes during stimulus exposure, outside of predetermined categories. Specifically, previous research has shown that emotional expression is reflected on various levels of the language structure, including the descriptive meaning (i.e., aspects expressed by a linguistic word or formulation) and the connotative meaning (i.e., the emotions implied by linguistic features, such as the chosen synonyms or taxonomy; Majid 2012). So far, research on body‐related emotions contained in spoken language remains scarce. Notably, studies examining concomitant features of language expression during mirror exposure have reported an increase in fundamental frequency (f0), as a marker of vocally encoded emotional arousal, during mirror exposure, suggesting that it might be influenced by experienced distress (Baur et al. 2020; Opladen et al. 2024). However, to date, no study has examined the semantic content of spoken language during mirror exposure, which constitutes both a critical proxy of momentary emotion and a potential complement to self‐report measures (Mauss and Robinson 2009).

Bottom‐up semantic analyses of spoken language require multi‐layered analyses of large, complex datasets. Recent advances in machine learning (ML) and natural language processing (NLP) have enabled the automated analysis of texts, for example social media posts or interviews, to detect indicators of mental health conditions (Jiang et al. 2020; Zhang et al. 2022), including depression (Aragón et al. 2023) and ED (Benitez‐Andrades et al. 2021; López‐Úbeda et al. 2019; Wang 2021). Briefly, the use of ML and NLP to date can be classified into three broader categories that is, classification, topic modeling, and sentiment analysis (Merhbene et al. 2024). Overall, studies focused on ED have thus far employed different ML approaches in line with their varying aims, and so forth, identifying whether tweets were written by people with ED (Benitez‐Andrades et al. 2021) or identifying early signs of anorexia nervosa (Paul et al. 2018). One ML approach is *Bidirectional Encoder Representations from Transformers* (BERT; Devlin et al. 2019), which allows for the identification of emotional states based on linguistic expression, conveying relationships between words and phrases as well as context (Eberhardt et al. 2024; Lalk et al. 2025). Moreover, the *Robustly Optimized BERT Approach* (RoBERTa), a refined version of BERT, shows improved performance by enhanced contextual understanding (Liu et al. 2019). Using RoBERTa, Eberhardt et al. (2024) successfully predicted emotional states based on transcripts of patients' speech during cognitive‐behavioral therapy sessions, showing positive correlations with patient‐reported positive emotions and negative correlations with negative self‐reported emotions. Additionally, comparing different BERT models in the field of ED, Benitez‐Andrades et al. (2021) found that RoBERTa performed especially well in identifying whether tweets were written by people with or without eating disorders, warranting the recommendation of this model when analyzing transcribed spoken language. Furthermore, recent research employing NLP to analyze typical terms and linguistic characteristics of women with anorexia nervosa (AN) found that the models correctly differentiated between individuals with and without AN (Maćkowska et al. 2024). So far, however, no study has focused on ML‐based analysis of verbally expressed responses of women with ED during mirror exposure.

To address the aforementioned research gaps, the present study examined verbally expressed emotional experiences in women with and without ED, differentiating both negative and positive emotions. To this end, participants underwent a three‐minute mirror task, looking at their reflection while wearing neutral underwear and freely describing their body. Transcripts were analyzed using a state‐of‐the‐art RoBERTa‐based NLP model, computing the probabilities of occurrence of a range of positive and negative emotions and relating them to ED symptom severity.

Given previous findings that women with AN (Vocks et al. 2007) and BN (Trentowska et al. 2017) reported experiencing more negative emotions when viewing their own bodies in a mirror, as assessed via self‐report questionnaires, we hypothesized that women with AN and BN would express significantly more negative emotions than healthy women, as based on the emotional content analysis of their verbal transcripts. Furthermore, following previous evidence that women with eating disorders show blunted or reduced positive emotional responses when confronted with images of bodies (Vocks et al. 2010), we hypothesized that women with AN or BN would express significantly fewer positive emotions than healthy women. Further, given prior findings that negative emotions are positively associated with eating disorder symptom severity (e.g., Espeset et al. 2012; Kot et al. 2021), we hypothesized that across participants, a higher relative likelihood of negative emotions would correlate positively with symptom severity, while a higher likelihood of positive emotions would correlate negatively with symptom severity. Finally, to further clarify the role of specific emotions, we conducted an exploratory analysis to examine which individual emotions were the strongest predictors of symptom severity when controlling for the presence of an eating disorder diagnosis.

## 2 Method

### 2.1 Sample and Recruitment

The sample was recruited as part of a larger research project (Tanck et al. 2021, 2022). Inclusion criteria for the present study were: (1) female gender and (2) age between 18 and 45 years. Exclusion criteria were: (1) current pregnancy, (2) current alcohol and/or substance abuse, and (3) acute suicidal tendencies or self‐harming behavior. Women with AN or BN were recruited in psychosomatic clinics in northern Germany, and were invited to participate during their inpatient stay. Healthy participants were recruited via flyers and e‐mail listings. After participating, the women received course credit or a payment of €25 as reimbursement. All participants provided informed consent before participation. The study was approved by the ethics committee of Osnabrück University.

### 2.2 Measures

#### 2.2.1 Eating Disorder Examination‐Questionnaire

To measure ED symptom severity, we used the global score of the Eating Disorder Examination‐Questionnaire (EDE‐Q; Fairburn and Cooper 1993; German Version: Hilbert et al. 2019). The EDE‐Q is a 22‐item self‐report questionnaire comprising four subscales, that is, “Restraint”, “Eating concern”, “Shape concern”, and “Weight concern”. Items are rated a 7‐point Likert scale ranging from 0 (*no days/none of the times/not at all*) to 6 (*every day/every time/markedly*). Internal consistency in the present sample was good (Cronbach's *α* = 0.81).

#### 2.2.2 Audio File Recording and Transcription

Audio files were created for each woman's responses during the three‐minute mirror task and subsequently transcribed into text form. *N* = 132 transcripts were included in this analysis. Spoken language was processed in sentence form; to be considered as a sentence, a minimum of three words was required. The word count of the transcripts ranged from 16 to 802 words, with an average of *M* = 284.89 (SD = 116.96) words per transcript.

### 2.3 Procedure

As previously described (Tanck et al. 2021, 2022), upon arrival, participants received the study information and provided written informed consent. Study eligibility was then rechecked, with the presence of an AN or BN diagnosis being validated using the *Structured Clinical Interview* (SCID‐IV) for Axis I disorders of the *Diagnostic and Statistical Manual of Mental Disorders, Fourth Edition* (DSM‐IV; Wittchen et al. 1997). Participants were then asked to complete a questionnaire battery, including the EDE‐Q. Next, participants were asked to undress down to their underwear set and stand in a mirror booth consisting of three mirrors positioned orthogonally to each other. This set‐up provided participants with an all‐around view of their bodies. During the following three‐minute period, participants were asked to describe their body and how they currently feel about it. No therapeutic guidance was provided. Participants' descriptions were recorded as audio files. Upon task completion, participants were debriefed and compensated.

### 2.4 Machine Learning Analysis

#### 2.4.1 RoBERTa and the *GermanEmotions* Dataset

Based on participants' transcripts, emotion classification was performed using BERT models (Grootendorst 2022). These models are further developments of one‐way language models that analyze a text by “reading” it in one direction (e.g., from left to right). BERT analyzes text bidirectionally by considering neighboring words, thus leading to a better contextual understanding (Devlin et al. 2019).

To conduct the emotion analysis, we used the *GermanEmotions* model by Lalk et al. (2025) as our inference model, which the authors trained on a translated version of the *GoEmotions* dataset (Demszky et al. 2020). The original *GoEmotions* dataset was based on the analysis of 54 k comments from the social media platform Reddit, while excluding offensive, vulgar, religion, and identity words. These comments were originally coded by three raters, using a list of 27 emotions plus one neutral category multiple selection was possible, that is, several emotions could be assigned to one sentence. Lalk et al. (2025) used an *XLM‐RoBERTa‐base* model (Conneau et al. 2019) as the basis for fine‐tuning. The final model showed similar metrics (F1_macro_ = 0.45, Kappa_macro_ = 0.42, *Accuracy* = 0.41; Lalk et al. 2025) to the original English‐language dataset (F1_macro_ = 0.46, Demszky et al. 2020) and moderate agreement of the predicted and labeled emotions (Cohen's Kappa = 0.42; see Lalk et al. 2025, for more detailed information).

**TABLE 1:** Sample characteristics.

	Anorexia nervosa (*n* = 36)		Bulimia nervosa (*n* = 24)		Healthy women (*n* = 72)	
	*M*	*SD*	*M*	*SD*	*M*	*SD*
Body mass index (in kg/m^2^)	16.99	2.08	21.75	3.03	21.23	2.81
Age (in years)	26.47	7.60	23.00	22.00	23.11	3.20
EDE‐Q restraint	3.42	1.80	3.67	1.86	1.06	1.03
EDE‐Q eating concern	3.28	1.23	3.73	1.44	0.47	0.60
EDE‐Q shape concern	4.60	1.12	4.76	1.15	1.44	0.97
EDE‐Q weight concern	3.91	1.23	3.73	1.44	1.05	0.95
EDE‐Q global score	3.90	1.18	4.27	1.21	1.10	0.77

*Note*: Eating Disorder Examination‐Questionnaire (EDE‐Q) subscales and global score.

This pre‐trained model was applied by running each sentence of the transcript through the model. In this way, the classification of emotions was evaluated as a probability, meaning that the presence of each of the 28 emotions in a sentence was rated between 0 and 1. For example, a probability of 0.85 for *anger* and 0.10 for *sadness* indicated that a sentence may be classified simultaneously within both emotions, with different probabilities of this emotion being present. Consequently, the probabilities of all 28 emotions would cumulate to 1 for each sentence. Finally, by calculating means of the probabilities of the expressed emotions across all sentences of the transcript, we determined a mean probability for all emotions for the corresponding transcript per participant.

#### 2.4.2 Analysis of Group Differences in Expressed Emotions in Anorexia Nervosa, Bulimia Nervosa, and Healthy Women

Group differences in the frequency of expressed emotions were analyzed using one‐way ANOVAs with Group (AN, BN, and healthy women) as the between‐subjects factor. To control for multiple testing across the different emotions, *p*‐values were adjusted using the Benjamini–Hochberg procedure. For ANOVAs that reached significance, pairwise post hoc *t*‐tests (with pooled error variance) were conducted between groups, and their *p*‐values were locally corrected using the Benjamini–Hochberg procedure. As measures of effect size, eta squared (*η* ^2^) is reported for ANOVAs and Cohen's *d* for post hoc comparisons. The significance level was set at *α* < 0.05 (two‐tailed).

#### 2.4.3 Prediction of Eating Disorder Symptom Severity by the Extent of Negative and Positive Emotions

**FIGURE 1:**
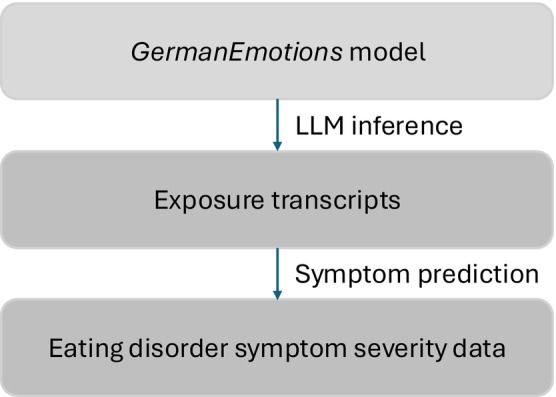
Workflow of this paper. The *GermanEmotions* model (Lalk et al. 2025) was applied for inference on the exposure transcripts and the inferred emotion features were then employed to predict eating disorder symptom severity. Additional features were word count and diagnostic status in a sensitivity analysis.

As independent variables, we computed the mean probabilities of occurrence of the inferred features (i.e., 27 emotions and a “neutral” feature) for each participant and the mean word count (i.e., the number of words) of transcripts, amounting to 29 features. The dependent variable was self‐reported symptom severity on the EDE‐Q (Hilbert et al. 2019). Subsequently, we conducted an equivalent sensitivity analysis, in which diagnostic status (i.e., women with vs. without ED) was included in the model as an additional predictor to elucidate which emotions continued to have an impact on symptom severity after considering the presence of an ED diagnosis. For an overview of the workflow of this paper, see Figure 1.

##### 2.4.3.1 Model Evaluation and Machine Learning Algorithms

The model evaluation was evaluated using a nested cross‐validation procedure with various machine learning algorithms (*XRAI*; Lalk and Mathew 2024). Random and unstratified data splits were used to optimize the model via inner and outer loops. For the initial selection of the model and the optimization of the hyperparameters, five inner loops were utilized first, followed by 10 outer loops, which were then used to validate the selected model in the respective test fold. This ensured that each test fold was predicted only once and not multiple times by each model, thus preventing overfitting. To assess the accuracy of the model, the correlation (*r*), the normalized root mean squared error (NRMSE), and the mean absolute error (MAE) were considered. Mean values and 95% confidence intervals were calculated during the outer loops for all metrics using bootstrapping. When running through the inner loops, six different algorithms were tested against each other for each data split, and the most suitable was used for the estimation of the respective test loop. This means that various algorithms may be used in the external loops. The following algorithms were tested against each other: (1) *Least absolute shrinkage and selection operator* (LASSO; Tibshirani 1996), (2) *Elastic net regularization and variable selection* (Elastic Net; Zou and Hastie 2005), (3) *eXtreme Gradient Boosting* (XGBoost; Chen and Guestrin 2016), (4) *Random Forest* (RF; Breiman 2001), (5) *Support Vector Regression* (SVR; Cortes and Vapnik 1995), and (6) *SuperLearner* (van der Laan et al. 2007). The *SuperLearner* used all of the predicted outcomes of the previous five learners as features for an SVR meta‐learner. Altogether, therefore, six models competed in the internal cross‐validation. For more details, see (Lalk, Steinbrenner, Kania, et al. 2024).

##### 2.4.3.2 Model Explanation Using Shapley Values

To determine the importance of different emotions within the prediction, we used the Python package *SHapley Additive exPlanations* (SHAP; Lundberg et al. 2019; Lundberg and Lee 2017) to investigate the influence of the emotions on the model outcome (i.e., symptom severity). Shapley values represent the influence of a single feature by removing or adding it from different combinations of features and subsequently measuring the change in model prediction (Shapley 1951). They allow for the calculation of a relative SHAP value for each feature, assessing each feature's relative importance (Lalk et al. 2025; Lalk, Steinbrenner, Kania, et al. 2024; Lalk, Steinbrenner, Pena, et al. 2024). To enable significance testing, each feature value was correlated with its respective SHAP values for each of the 10 outer cross‐validation folds, resulting in 10 correlation coefficients per feature. This was done only once per test fold, as each external test fold was predicted only once, but could contain different model predictions if more than one model was selected in the external cross‐validation. By implementing bootstrapping, we calculated *z*‐values and *p*‐values for each feature, which were adjusted using the Benjamini–Hochberg procedure with a false discovery rate of 5%.

## 3 Results

### 3.1 Sample Characteristics and Transcript Coding

The sample consisted of healthy women (HC; *n* = 72), women with anorexia nervosa (AN; *n* = 36), and women with bulimia nervosa (BN; *n* = 24). Further sample characteristics are presented in Table 1.

In the following, we provide three randomly chosen sentences from the analyzed transcripts as well as their coding by the NLP with regard to the emotions present. The sentences were coded considering all 28 emotions. Each emotion has a probability of occurrence ranging between 0 and 1 in a respective sentence, therefore amounting to 1. As all sentences were in German, we have translated the sentences into English here for ease of understanding.

The sentence “Yes, I like my body.” was coded as approval (0.469), admiration (0.244), love (0.200), joy (0.042), neutral (0.015), pride (0.008), excitement (0.007), desire (0.003), optimism and amusement (both 0.002), disappointment, disgust, embarrassment, gratitude, relief, sadness, and surprise (all 0.001), anger, annoyance, caring, confusion, curiosity disapproval, fear, grief, nervousness, realization, and remorse (all < 0.001).

The sentence “I'm really ashamed of my legs, my thighs and lower legs, my hips; yes, then there's this unpleasant feeling when I look at my stomach, because I think it's bloated and that bothers me and it kind of disgusts me to see my body like that.” was coded as embarrassment (0.786), disgust (0.154), annoyance (0.014), disappointment (0.009), anger and sadness (0.007), neutral (0.005), disapproval, nervousness, and remorse (0.003), fear and realization (both 0.002), amusement, confusion, love, and pride (all 0.001), admiration, approval, caring, curiosity, desire, excitement, gratitude, grief, joy, optimism, relief, and surprise (all < 0.001).

The sentence “I don't really know what to say about it, as I'm very anxious and also a bit dissatisfied.” was coded as nervousness (0.532), disappointment (0.285), sadness (0.041), annoyance (0.037), confusion (0.031), fear (0.013), anger (0.012), embarrassment (0.007), approval (0.006), disgust (0.005), desire, optimism, and realization (all 0.004), caring, curiosity, disapproval, excitement, joy, remorse (all 0.002), admiration, grief, love, relief, and neutral (all 0.001), amusement, gratitude, pride, and surprise (all < 0.001).

### 3.2 Analysis of Group Differences in Expressed Emotions in Anorexia Nervosa, Bulimia Nervosa, and Healthy Women

Mean probabilities, standard deviations, *p*‐values, and effect sizes of the ANOVAs comparing the three groups (AN vs. BN vs. HC) are presented in Table 2.

**TABLE 2:** Mean relative probabilities (*M*), standard deviations (*SD*) and ANOVA values.

Emotions	AN (*n* = 36)		BN (*n* = 24)		HC (*n* = 72)		*F*	*df*_1_	*df*_2_	*p*	*η* ^*2*^	*p*_adj_
	*M*	*SD*	*M*	*SD*	*M*	*SD*						
**Negative**	0.040	0.018	0.035	0.015	0.018	0.012	33.101	2	129	< 0.001***	0.339	< 0.001***
Anger	0.016	0.014	0.030	0.003	0.009	0.006	6.935	2	129	0.001**	0.097	0.003**
Annoyance	0.049	0.028	0.037	0.037	0.044	0.044	1.162	2	129	0.316	0.018	0.421
Confusion	0.032	0.017	0.036	0.030	0.018	0.054	0.795	2	129	0.454	0.012	0.529
Disappointment	0.064	0.045	0.060	0.028	0.055	0.027	10.025	2	129	< 0.001***	0.135	< 0.001***
Disapproval	0.045	0.063	0.041	0.059	0.061	0.077	0.609	2	129	0.545	0.009	0.587
Disgust	0.088	0.112	0.151	0.016	0.075	0.019	8.61	2	129	< 0.001***	0.118	0.001**
Embarrassment	0.037	0.026	0.071	0.010	0.031	0.012	6.544	2	129	0.002**	0.092	0.004**
Fear	0.034	0.047	0.058	0.003	0.019	0.004	8.977	2	129	< 0.001***	0.122	0.001**
Grief	0.001	0.001	0.001	0.000	0.001	0.000	15.163	2	129	< 0.001***	0.190	< 0.001***
Nervousness	0.034	0.033	0.042	0.008	0.028	0.012	12.574	2	129	< 0.001***	0.163	< 0.001***
Remorse	0.004	0.001	0.006	0.001	0.003	0.001	7.744	2	129	0.001**	0.107	0.002**
Sadness	0.080	0.098	0.081	0.021	0.075	0.028	13.428	2	129	< 0.001***	0.172	< 0.001***
**Positive**	0.015	0.008	0.019	0.008	0.030	0.010	42.089	2	129	< 0.001***	0.395	< 0.001***
Admiration	0.012	0.054	0.016	0.037	0.037	0.038	5.712	2	129	0.004**	0.081	0.008*
Amusement	0.004	0.003	0.006	0.006	0.002	0.016	0.927	2	129	0.398	0.014	0.507
Approval	0.060	0.058	0.056	0.126	0.069	0.076	14.139	2	129	< 0.001***	0.180	< 0.001***
Caring	0.003	0.001	0.004	0.003	0.002	0.004	2.266	2	129	0.108	0.034	0.189
Curiosity	0.013	0.009	0.023	0.007	0.006	0.016	1.545	2	129	0.217	0.023	0.329
Desire	0.008	0.011	0.019	0.018	0.008	0.034	2.057	2	129	0.132	0.031	0.217
Excitement	0.005	0.011	0.008	0.010	0.007	0.023	0.807	2	129	0.448	0.012	0.529
Gratitude	0.001	0.007	0.001	0.003	0.003	0.006	1.464	2	129	0.235	0.022	0.329
Joy	0.012	0.040	0.023	0.080	0.027	0.078	17.235	2	129	< 0.001***	0.211	< 0.001***
Love	0.016	0.047	0.025	0.068	0.040	0.084	7.345	2	129	< 0.001***	0.102	0.002**
Optimism	0.009	0.009	0.015	0.021	0.008	0.022	7.411	2	129	< 0.001***	0.103	0.002**
Pride	0.002	0.010	0.003	0.007	0.006	0.008	5.865	2	129	0.004**	0.083	0.007*
Realization	0.065	0.067	0.072	0.059	0.065	0.054	0.168	2	129	0.846	0.003	0.846
Relief	0.004	0.018	0.009	0.007	0.009	0.009	1.477	2	129	0.232	0.022	0.329
Surprise	0.007	0.010	0.015	0.005	0.004	0.014	0.337	2	129	0.715	0.005	0.741
**Neutral**	0.294	0.144	0.170	0.326	0.288	0.159	0.755	2	129	0.472	0.012	0.529

*Note*: AN = anorexia nervosa, BN = bulimia nervosa, HC = healthy controls, that is, women without eating disorders; significance levels * < 0.05, ** < 0.005, *** < 0.001; *p*_*adj*_ = Benjamini–Hochberg adjustment.

The ANOVA revealed significant group differences in negative emotions. Post hoc comparisons indicated that women with AN and BN did not differ from each other, while both women with AN and BN reported significantly higher negative emotions compared to HC (AN vs. HC: *t*(129) = 7.60, *p* = < 0.001, *d* = 1.55; BN vs. HC: *t*(129) = 4.98, *p* = < 0.001, *d* = 1.17). Regarding positive emotions, the ANOVA also revealed significant group differences, and post hoc comparisons showed that all three groups differed significantly from each other (AN < BN < HC; AN vs. BN: *t*(129) = −2.05, *p* = 0.04, *d* = −0.54; AN vs. HC: *t*(129) = −8.72, *p* = < 0.001, *d* = −1.78; BN vs. HC: *t*(129) = −5.25, *p* = < 0.001, *d* = −1.24). No significant group differences were found regarding neutral emotions.

Among the negative emotions, the ANOVAs revealed significant group differences in disappointment, grief, nervousness, sadness, anger, disgust, embarrassment, remorse, and fear, and no significant group differences in annoyance, confusion, and disapproval. The ANOVA results are presented in Table 2. Regarding disappointment, post hoc comparisons indicated no significant differences between AN and BN, while both AN and BN differed significantly from HC (AN > BN > HC; AN vs. HC: *t*(129) = 4.21, *p* < 0.001, *d* = 0.85; BN vs. HC: *t*(129) = 2.68, *p* = 0.012, *d* = 0.63). Regarding grief, post hoc comparisons indicated significant differences between AN and BN (*t*(129) = 2.09, *p* = 0.039, *d* = 0.55), between AN and HC (*t*(129) = 5.44, *p* < 0.001, *d* = 1.11), and between BN and HC (*t*(129) = 2.38, *p* = 0.019, *d* = 0.56). Regarding nervousness, post hoc comparisons indicated significant differences between AN and HC (*t*(129) = 4.67, *p* < 0.001, *d* = 0.95) as well as between BN and HC (*t*(129) = 3.11, *p* = 0.003, *d* = 0.73), and no significant differences between AN and BN. Regarding sadness, post hoc comparisons revealed significant differences between AN and HC (*t*(129) = 4.58, *p* < 0.001, *d* = 0.94) and between BN and HC (*t*(129) = 3.64, *p* = 0.001, *d* = 0.86), and no significant differences between AN and BN. Regarding anger, post hoc comparisons showed significant differences between AN and HC (*t*(129) = 3.69, *p* = 0.001, *d* = 0.75) and no significant differences between AN and BN or between BN and HC. Regarding disgust, post hoc comparisons indicated significant differences between AN and HC (*t*(129) = 3.80, *p* = 0.001, *d* = 0.78) and between BN and HC (*t*(129) = 2.69, *p* = 0.012, *d* = 0.63), and no significant differences between AN and BN. Regarding embarrassment, post hoc comparisons revealed significant differences between AN and HC (*t*(129) = 3.36, *p* = 0.003, *d* = 0.69) and between BN and HC (*t*(129) = 2.24, *p* = 0.040, *d* = 0.53). Regarding remorse, post hoc comparisons indicated significant differences between AN and HC (*t*(129) = 3.89, *p* < 0.001, *d* = 0.79) and no significant differences between AN and BN or between BN and HC. Regarding fear, post hoc comparisons showed significant differences between AN and HC (*t*(129) = 4.17, *p* < 0.001, *d* = 0.84) and no significant differences between AN and BN or between BN and HC.

Conversely, the ANOVAs of specific positive emotions revealed significant group differences in admiration, approval, joy, love, optimism, and pride. There were no significant group differences for amusement, caring, curiosity, desire, excitement, gratitude, realization, relief, and surprise. All ANOVA results are presented in Table 2. Regarding admiration, post hoc comparisons indicated significant differences between AN and BN (*t*(129) = −2.48, *p* = 0.021, *d* = −0.66) as well as between AN and HC (*t*(129) = −3.25, *p* = 0.004, *d* = −0.66), and no significant differences between BN and HC. Regarding approval, post hoc comparisons showed significant differences between AN and HC (*t*(129) = −4.80, *p* < 0.001, *d* = −0.98) as well as between BN and HC (*t*(129) = −3.57, *p* = 0.001, *d* = −0.84), and no significant differences between AN and BN. Regarding joy, post hoc comparisons revealed significant differences between AN and HC (*t*(129) = −5.46, *p* < 0.001, *d* = −1.12) as well as between BN and HC (*t*(129) = −3.64, *p* = 0.001, *d* = −0.86), and no significant differences between AN and BN. Regarding love, post hoc comparisons showed significant differences between AN and HC (*t*(129) = −3.77, *p* = 0.001, *d* = −0.77) and no significant differences between AN and BN or between BN and HC. Regarding optimism, post hoc comparisons indicated significant differences between AN and HC (*t*(129) = −3.12, *p* = 0.004, *d* = −0.64) as well as between BN and HC (*t*(129) = −3.06, *p* = 0.004, *d* = −0.72), and no significant differences between AN and BN. Regarding pride, post hoc comparisons revealed significant differences between AN and HC (*t*(129) = −3.42, *p* = 0.003, *d* = −0.70) and no significant differences between AN and BN or between BN and HC.

### 3.3 Prediction of Eating Disorder Symptom Severity by the Extent of Negative and Positive Emotions

**FIGURE 2:**
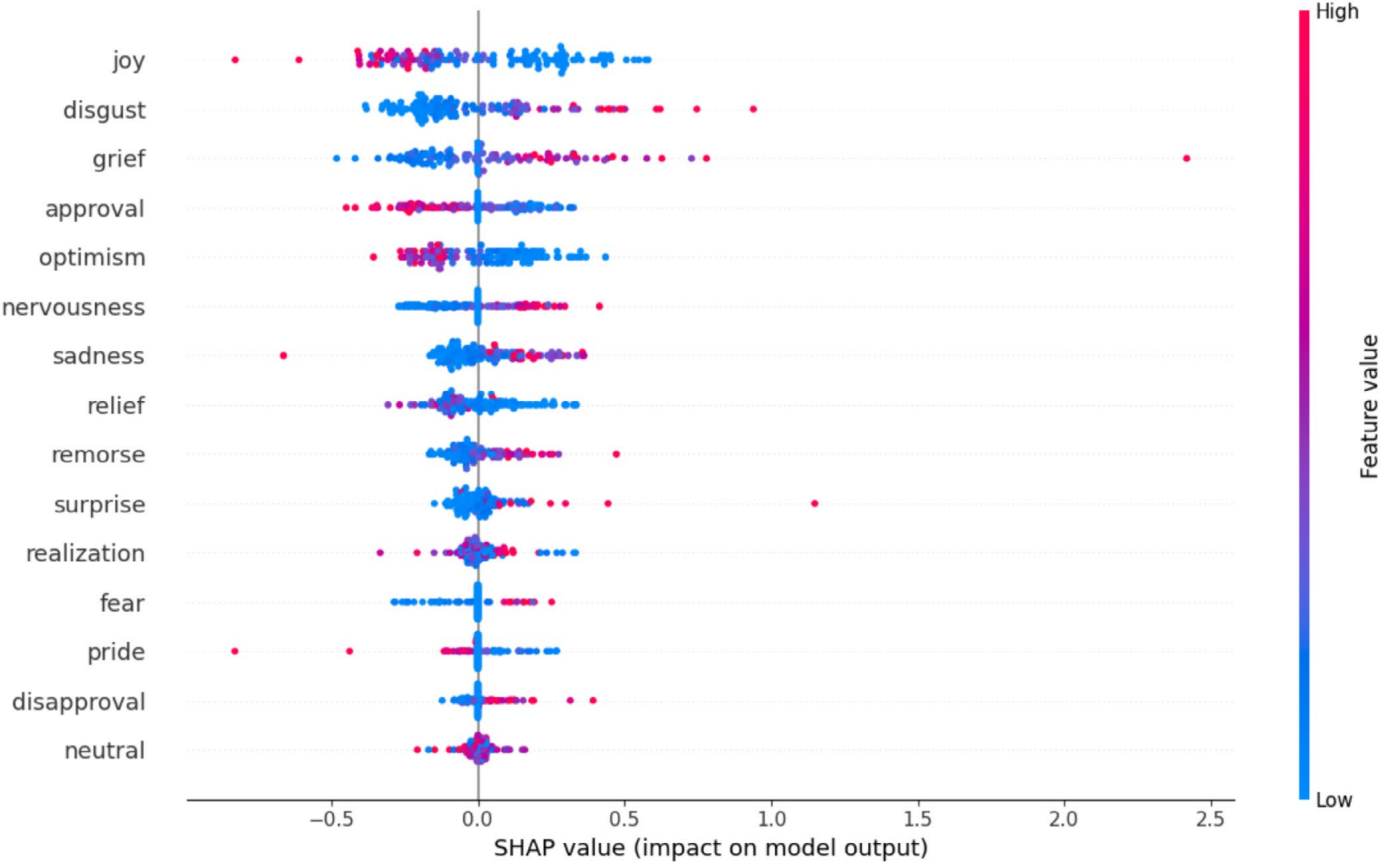
Relative influence of the top 15 emotions on eating disorder symptom severity (as measured by the Eating Disorder Examination‐Questionnaire; EDE‐Q). Red dots indicate a higher probability of occurrence of the emotions; blue dots indicate a lower probability of occurrence; purple dots indicate a moderate probability of occurence of the emotions. The x‐axis illustrates the influence of the emotions on the prediction of eating disorder symptoms (EDE‐Q). Each dot refers to one transcript. Here, the presence of a diagnosis is not included as a predictor.

The relative SHAP value of the extracted emotions regarding the influence on ED symptom severity, as measured by the EDE‐Q global score (Aardoom et al. 2012), correlations of the emotions with the SHAP value, and *p*‐values across both groups are presented in Table 3 and Figure 2. For the prediction of ED symptom severity, the model with 28 emotions and word count, without considering the presence of an ED diagnosis as a predictor, showed a performance of *r =* 0.57 [95%‐CI: 0.48, 0.67]; NRMSE = 0.83 [95%‐CI: 0.78, 0.88]; MAE = 1.20 [95%‐CI: 1.08, 1.31]. In the outer loops, *Random Forest* was selected seven times as the best algorithm and used for the calculation, and *Support Vector Regression* (SVR) three times. The respective hyperparameters are reported in Table S5. The emotions that showed the strongest contribution to change in ED symptom severity, without considering the presence of an ED diagnosis, were *joy* (*r* = −0.80, *p* < 0.001; relative SHAP value 14.46%), *disgust* (*r* = 0.87, *p* < 0.001; relative SHAP value 11.88%), *grief* (*r* = 0.79, *p* < 0.001; relative SHAP value 11.37%), *approval* (*r* = −0.79, *p* < 0.001; relative SHAP value 8.87%), and *optimism* (*r* = −0.80, *p* < 0.001; relative SHAP value 8.69%).

**TABLE 3:** Prediction of eating disorder symptom severity: Emotions and Word Count, Relative SHAP value, and correlations with SHAP value.

Emotions	Eating Disorder Examination‐Questionnaire								
	Without ED diagnosis as a predictor					With ED diagnosis as a predictor			
	RSV	*r*	*p*	*p*_adj_		RSV	*r*	*p*	*p*_adj_
					Diagnosis	81.91%	> −0.99	< 0.001***	< 0.001***
Joy	14.46%	−0.80	< 0.001***	< 0.001***	Disgust	3.27%	0.96	< 0.001***	< 0.001***
Disgust	11.88%	0.87	< 0.001***	< 0.001***	Caring	2.54%	0.18	0.038*	0.188
Grief	11.37%	0.79	< 0.001***	< 0.001***	Optimism	2.21%	−0.26	0.017*	0.152
Approval	8.87%	−0.79	< 0.001***	< 0.001***	Sadness	1.88%	−0.27	0.054	0.188
Optimism	8.67%	−0.80	< 0.001***	< 0.001***	Relief	1.08%	−0.20	0.073	0.206
Nervousness	6.84%	0.66	< 0.001***	< 0.001***	Surprise	0.80%	0.24	0.061	0.188
Sadness	6.26%	0.56	0.002**	0.004**	Words	0.75%	0.11	0.099	0.235
Relief	6.15%	−0.50	< 0.001***	< 0.001***	Anger	0.73%	0.14	0.051	0.188
Remorse	4.45%	0.76	< 0.001***	< 0.001***	Approval	0.71%	−0.21	0.057	0.188
Surprise	3.76%	0.65	< 0.001***	< 0.001***	Joy	0.64%	−0.22	0.024*	0.151
Realization	3.02%	0.23	0.154	0.243	Realization	0.60%	−0.09	0.457	0.650
Fear	2.44%	0.21	0.047	0.100	Pride	0.53%	−0.04	0.630	0.781
Pride	2.42%	−0.30	0.013	0.034	Embarrassment	0.48%	0.07	0.503	0.650
Disapproval	2.14%	0.52	1	1	Neutral	0.45%	−0.08	0.498	0.650
Neutral	1.75%	−0.20	0.195	0.292	Disappointment	0.41%	−0.21	0.024	0.369
Anger	1.43%	0.16	0.121	0.213	Remorse	0.35%	−0.11	0.424	0.650
Love	1.42%	−0.17	0.116	0.213	Fear	0.32%	0.18	0.080	0.207
Confusion	1.23%	−0.25	0.046	0.100	Disapproval	0.16%	0.10	0.024*	0.369
Desire	0.38%	−0.11	0.128	0.213	Grief	0.07%	0.08	0.285	0.509
Embarrassment	0.37%	0.17	0.110	0.213	Nervousness	0.06%	0.04	0.302	0.509
Words	0.36%	−0.09	0.277	0.394	Desire	0.04%	0.04	0.283	0.509
Annoyance	0.23%	0.05	0.302	0.392	Confusion	0%	0.01	0.787	0.938
Excitement	0.09%	0.08	0.291	0.392	Excitement	0%	−0.02	0.263	0.508
Admiration	0%	0	1	1	Love	0%	0.04	0.463	0.650
Amusement	0%	0	1	1	Admiration	0%	0	1	1
Caring	0%	0	1	1	Amusement	0%	0	1	1
Curiosity	0%	0	1	1	Annoyance	0%	0	1	1
Disappointment	0%	0	1	1	Curiosity	0%	0	1	1
Gratitude	0%	0	1	1	Gratitude	0%	0	1	1

*Note*: RSV = Relative SHAP Value that is, percentage contribution of emotion to the prediction of self‐reported symptom severity; *r* = Correlation with SHAP Value, significance levels of *p*‐values * < 0.05, ** < 0.005, *** < 0.001; *p*_adj_. = Benjamini–Hochberg adjustment for multiple testing of *p*‐value.

**FIGURE 3:**
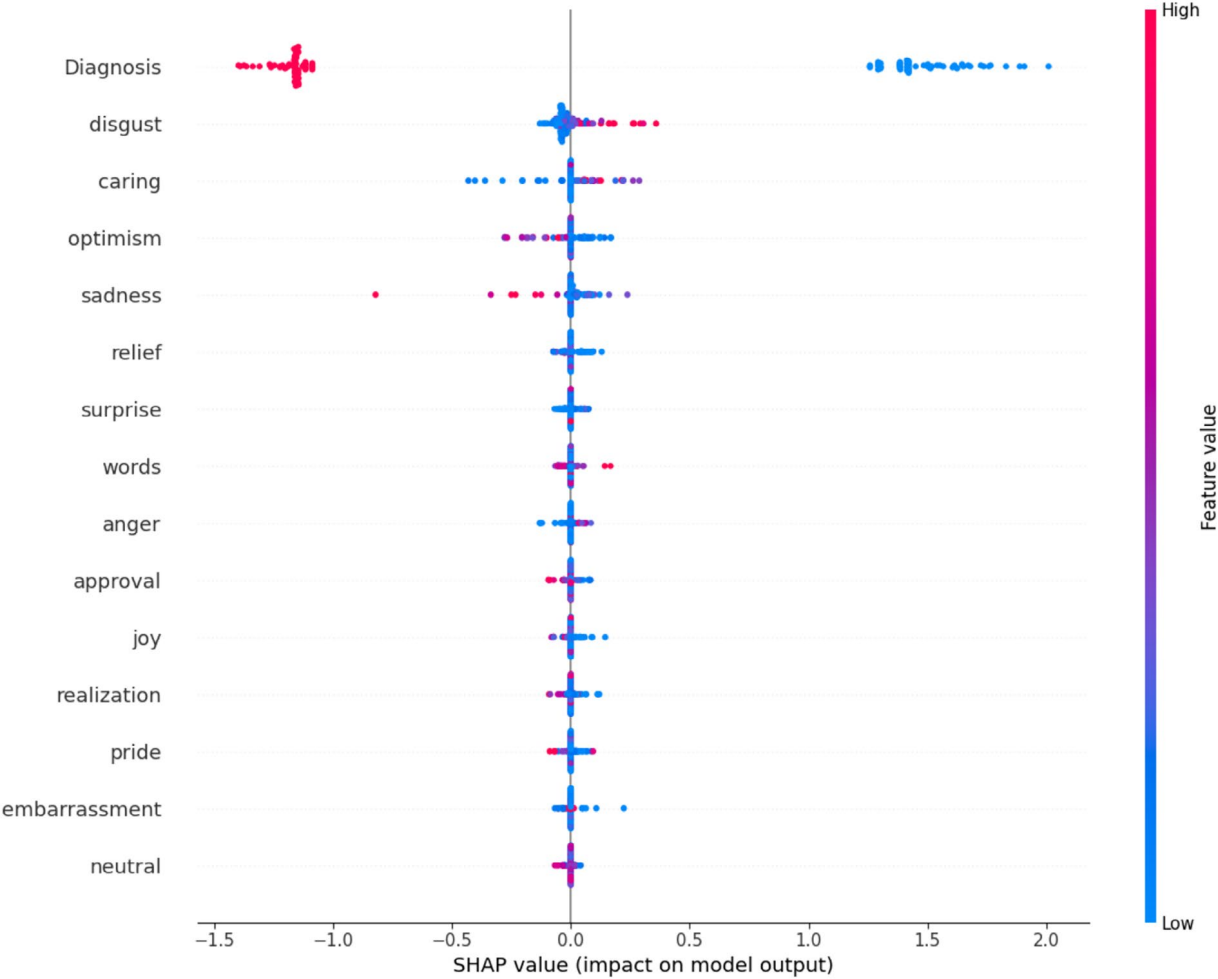
Relative influence of the top 15 emotions on eating disorder symptom severity (as measured by the Eating Disorder Examination‐Questionnaire; EDE‐Q). Red dots indicate a higher probability of occurrence of the emotions; blue dots indicate a lower probability of occurrence of the emotions; purple dots indicate a moderate probability of occurence of the emotions. The x‐axis illustrates the influence of the emotions on the prediction of eating disorder symptoms (EDE‐Q). Each dot refers to one transcript. Here, the presence of a diagnosis is included as a predictor. Of note, presence of an eating disorder diagnosis is coded as 0, and absence of an eating disorder diagnosis is coded as 1.

Figure 3 presents the relative SHAP value and correlation of the extracted emotions regarding the influence on ED symptom severity across both groups when considering the presence of an ED diagnosis as a predictor. The performance of the model improved to *r =* 0.82 [95%‐CI: 0.74, 0.89]; NRMSE = 0.59 [95%‐CI: 0.51, 0.70]; MAE = 0.84 [95%‐CI: 0.76, 0.92]. The selected algorithms were *LASSO* six times and *Random Forest* four times. The hyperparameters are listed in Table S6. The predictor ED diagnosis had the strongest impact on the model prediction (relative SHAP value 81.91%). Of the expressed emotions that differed significantly between women with and without ED, only *disgust* contributed significantly to the prediction of ED symptom severity beyond ED diagnosis (*r* = 0.96, *p* < 0.001; relative SHAP value 3.21%). When separating this analysis by models (i.e., LASSO vs. Random Forest; see Table S7), we found that essentially, LASSO only used ED diagnosis (relative SHAP value 97.2%) while Random Forest selected other variables that is, emotions as well and gave a lower value to ED diagnosis (relative SHAP value 68.9%).

## 4 Discussion

Using a state‐of‐the‐art NLP analysis approach, the present study aimed to extract occurring emotions from individuals' verbal expression, as recorded during a mirror task in women with AN and BN as well as healthy women. Specifically, we examined whether verbalized emotions differed between individuals with and without ED and explored whether specific emotions predicted ED symptom severity. Overall, results revealed that there were higher probabilities of negative emotions in women with AN or BN, versus healthy women. Regarding positive emotions, we further found lower probabilities of positive emotions in women with AN, versus BN, and in women with BN, versus healthy women, respectively. Moreover, across both groups, negative emotions were positively correlated with ED symptom severity and positive emotions were negatively correlated with ED symptom severity.

Regarding negative emotions associated with ED symptoms, we identified nine emotions—that is, sadness, grief, nervousness, disappointment, disgust, fear, remorse, embarrassment, and anger—that were more frequently reported by women with versus without an ED diagnosis. Critically, the present study is the first to identify these emotions during exposure to a disorder‐relevant stimulus that is, the mirror task. These findings extend previous research, which employed retrospective or category‐based emotional assessment and reported exacerbated body‐related guilt, shame, envy, embarrassment (Solomon‐Krakus et al. 2022), and self‐disgust (Kot et al. 2021) in women with ED. Using a bottom‐up approach, the present findings reveal a broader spectrum of negative emotions during the mirror task than previously assumed, thus informing emerging treatment approaches for AN and BN. Importantly, of all negative emotions identified above, disgust appears to be central, as it was the only emotion that still contributed significantly to the prediction of symptom severity after taking ED diagnosis into account as a predictor variable. Overall, these results align with previous research highlighting disgust as a central emotion in women with AN viewing themselves in the mirror (Kot et al. 2021). Furthermore, this finding may imply that disgust exerts an impact on disorder‐related symptoms across different groups rather than specifically affecting women with an ED diagnosis. Corroborating this hypothesis, recent studies indicate that individuals with AN or BN show higher disgust sensitivity (i.e., the extent to which individuals experience the feeling of disgust as unpleasant; Rabito‐Alcón et al. 2025). Moreover, research has shown that individuals with AN or BN display elevated self‐disgust (Bektas et al. 2022; Bell et al. 2017; Marques et al. 2021), that is, a feeling of disgust triggered by one's body, not as a consequence of an odor or taste stimulus, but by the internal evaluation of one's body or physical attributes as shameful (Powell et al. 2015). In their theoretical framework on the role of self‐disgust in ED psychopathology, Glashouwer and de Jong (2021) proposed that persistent self‐disgust after anorexia nervosa treatment may contribute to increased relapse risk, suggesting that targeting disgust could improve long‐term outcomes. In the present study, participants' verbal disgust‐related expressions were directed towards their own bodies, potentially reflecting self‐disgust and underscoring its central role during exposure to disorder‐relevant stimuli. However, as we did not assess specific subdimensions of disgust, firm conclusions about this association remain premature at present, warranting further investigation.

Relatedly, given that mirror exposure is a central component of ED treatment (Trentowska et al. 2014), our findings could yield important insights into why the effects of these interventions may be limited. Specifically, traditional exposure‐based models assume that symptom reduction in mirror exposure is achieved, inter alia, through habitation, thus requiring fear, a rapidly habituated emotion, to be predominant during mirror tasks in ED (Foa and Kozak 1986). However, the present findings demonstrate that disgust, as a less easily habituated emotion (Olatunji et al. 2009), is more frequently reported, which might explain the insufficient efficacy of mirror exposure in some individuals (Werthmann et al. 2025). Conversely, our results suggest that outcomes of mirror exposure might be improved through counterconditioning that is, by focusing on positively evaluated body parts or even increasing positive emotions (Griffen et al. 2018; Tanck et al. 2022).

Regarding positive emotions, women with ED expressed significantly less joy, approval, optimism, love, pride, and admiration when looking at their body than did women without ED. These findings are novel, as previous research, employing category‐based emotional assessment, merely demonstrated that women with AN or BN experienced fewer positive emotions than negative emotions compared to healthy women, without naming specifically occurring emotions (Tanck et al. 2021; Vocks et al. 2007). Our finding that women with AN or BN expressed significantly less joy is especially noteworthy given that a diminished experience of joy potentially stems from anhedonia (i.e., a reduced capacity to experience pleasure or positive affect), a common symptom in ED (Dolan et al. 2022). Furthermore, our finding that women with AN less frequently reported body‐related pride compared to women with BN and healthy women corresponds to a previous study that identified body pride as a predictor of higher well‐being in UK adults (Swami et al. 2018). Similarly, body‐related approval was less frequently expressed by women with AN or BN in our study, corresponding to previous research indicating that individuals with ED often experience difficulties with self‐compassion (Braun et al. 2016) and acceptance (Tylka and Wood‐Barcalow 2015). Admiration, that is, appreciating one's physical attributes, was also less prevalent in women with AN compared to healthy women and women with BN, potentially reflecting a distorted body perception and negative self‐evaluation. Importantly, this aligns with cognitive‐behavioral theories, which propose that negative self‐schemas related to body size and shape are maintaining factors in women with ED (e.g., Williamson et al. 2004). Overall, the diminished expression of positive emotions found among women with AN or BN, versus healthy women, in the present study might indicate a dearth of positive emotions in women with ED per se. Alternatively, this finding may also reflect potential difficulties in accepting positive emotions as well as a tendency to suppress them (Selby et al. 2019). Furthermore, this finding corroborates the growing understanding of body image as a multidimensional construct in which positive and negative dimensions may coexist independently (Overton et al. 2005). Accordingly, a positive body image is characterized not only by the absence of dissatisfaction but by the active embracing of positive feelings towards one's body (Tylka and Wood‐Barcalow 2015). At the same time, positive and negative body image may also be considered distinct constructs, with positive body image being associated with experiencing emotions related to the body, such as appreciation (e.g., appreciating the body for its health and functionality) and love (i.e., loving the body for what it is able to do, inner positivity; Tylka and Wood‐Barcalow 2015). In this regard, research has indicated that women with a positive body image report greater well‐being (Swami et al. 2018) and are more likely to engage in health‐promoting behaviors (Andrew et al. 2016). Thus, in view of the present findings, from a therapeutic perspective, rather than merely aiming to reduce negative emotions (Werthmann et al. 2025), it may be beneficial to foster the experience of positive emotions and the possibility to express them, for instance by focusing on positively evaluated body parts during mirror exposure (Tanck et al. 2021), or to combine positively experienced body‐focused activities.

Some limitations of the present study should be acknowledged. Regarding limitations of the machine learning approach, first, the small sample size limited the statistical power despite the use of nested cross‐validation (Cearns et al. 2019). Second, as the model was trained on Reddit data (*GoEmotions* dataset), caution is warranted when transferring the findings to clinical populations, for which data from tasks designed for clinical assessment may be more appropriate (Cearns et al. 2019). Third, the brevity of the transcripts from the mirror task may have limited the linguistic richness. Finally, the model may not have perfectly distinguished between related emotions, and due to the study's focus on linguistic content alone, the role of nonverbal cues was neglected. Future research should integrate multimodal data for a more complete assessment of emotions. Although efforts were made to mitigate the limitations within the machine learning process, several factors related to the study population and procedures also warrant consideration: The accuracy of emotion extraction is inherently dependent on participants' ability to accurately identify and articulate their feelings, and variability in individuals' capacity to differentiate between nuanced emotional states, such as fear and disgust, may have influenced the findings. Furthermore, the current study was limited to female participants, further featuring clinical groups undergoing inpatient treatment at the time of data collection, and healthy participants from the general population. Relatedly, the ED sample did not include women with diagnoses beyond AN or BN, that is, binge eating disorder or EDNOS. Additionally, potential comorbidities could not be included in the present analyses, precluding any conclusions regarding their influence on emotional states. Thus, to enhance generalizability, future research should include a more diverse sample, incorporating male participants and individuals with a broader range of sociodemographic, diagnostic and psychopathological characteristics, further reflecting them more broadly within analyses.

Relatedly, it should be noted that the present cross‐sectional findings do not permit causal conclusions. For instance, it is possible that BMI or body dissatisfaction contributed to negative emotions independently of the manipulation, thus requiring consideration in future research. Moreover, within the scope of this initial study, we focused on the EDE‐Q global score as the main measure of overall symptom severity (Aardoom et al. 2012). While we provide further in‐depth analyses for EDE‐Q subscales (i.e., restraint, eating concern, shape concern, and weight concern) in the Tables S1–S4, future research might further disentangle the association between subdimensions of body image and pertinent emotional states.

As the generalizability of the present findings is also limited by the specific context of the mirror task, further research should determine whether these body‐related emotions are similarly expressed in naturalistic social situations. Finally, given that the mirror task did not control for attentional focus during verbalization, it is possible that participants attended more readily to body parts that they evaluated as unattractive (Bauer et al. 2017). To attain a more comprehensive assessment, it would thus be beneficial to link these findings with measures of cognitive biases within future studies.

To the best of our knowledge, the present study is the first to examine body‐related negative and positive emotions in AN and BN, using a bottom‐up approach grounded in verbal transcripts during a mirror exposure task. Our findings provide new insights into meaningful associations between specific emotions and ED symptoms, further highlighting the unique role of disgust in predicting symptom severity. Notably, the findings have important implications for ED treatment, particularly regarding body exposure. Since disgust is less susceptible to habituation than fear (Olatunji et al. 2009), the effects of body exposure may be enhanced through an increased focus on positive emotions as part of counterconditioning, as such a focus appears to be more likely to bring about symptom improvement (Jansen et al. 2016; Tanck et al. 2022). Indeed, this is supported by evidence that individuals with AN or BN report significantly fewer positive emotions during mirror exposure compared to healthy women (Vocks et al. 2007). Therefore, incorporating strategies that enhance positive emotional experiences during body‐related interventions could improve treatment outcomes. Overall, integrating interventions specifically aimed at fostering positive emotions may be a promising pathway to help sustain treatment effects in individuals with AN or BN.

## Author Contributions

**Linda Marie Sadowski:** writing – original draft preparation, formal analysis, review and editing. **Christopher Lalk:** formal analysis, writing – review and editing. **Vanessa Hofschröer:** writing – review and editing. **Fanny Alexandra Dietel:** writing – original draft preparation, review and editing. **Julia Tanck:** investigation, writing – review and editing. **Julian A. Rubel:** writing – review and editing. **Andrea S. Hartmann:** conceptualization, writing – review and editing. **Silja Vocks:** conceptualization, supervision, writing – review and editing. All authors contributed to the compilation of the final manuscript and read and approved the submitted version.

## Funding

The authors have nothing to report.

## Conflicts of Interest

The authors declare no conflicts of interest.

## Supporting information


**Table S1:** Prediction of eating disorder symptom severity. Subscale “eating concern”: Emotions and word count, Relative SHAP value, and correlations with SHAP value.
**Table S2:** Prediction of eating disorder symptom severity. Subscale “restraint”: Emotions and word count, relative SHAP value, and correlations with SHAP value.
**Table S3:** Prediction of eating disorder symptom severity. Subscale “shape concern”: Emotions and word count, relative SHAP value, and correlations with SHAP value.
**Table S4:** Prediction of eating disorder symptom severity. Subscale “weight concern”: Emotions and word count, relative SHAP value, and correlations with SHAP value.
**Table S5:** Hyperparameters without including diagnosis as a predictor.
**Table S6:** Hyperparameters with diagnosis as a predictor.
**Table S7:** Feature importance correlation separated for RF and Lasso.

## Data Availability

The datasets generated for this study are available upon reasonable request to the corresponding first author.
