# Supplementary material for: Negative and Positive Body‐Related Emotions Derived From Voice Recordings During a Mirror Task in Anorexia and Bulimia Nervosa: A Natural Language Processing Approach Using RoBERTa
Source: Int J Eat Disord. 2025 Nov 26;59(3):546–59. doi: 10.1111/eat.70007 (PMC12979969; doi:10.1111/eat.70007)
Supplement: Supplementary file 1 — Table S1: Prediction of eating disorder symptom severity. Subscale “eating concern”: Emotions and word count, Relative SHAP value, and correlations with SHAP value. Table S2: Prediction of eating disorder symptom severity. Subscale “restraint”: Emotions and word count, relative SHAP value, and correlations with SHAP value. Table S3: Prediction of eating disorder symptom severity. Subscale “shape concern”: Emotions and word count, relative SHAP value, and correlations with SHAP value. Table S4: Prediction of eating disorder symptom severity. Subscale “weight concern”: Emotions and word count, relative SHAP value, and correlations with SHAP value. Table S5: Hyperparameters without including diagnosis as a predictor. Table S6: Hyperparameters with diagnosis as a predictor. Table S7: Feature importance correlation separated for RF and Lasso. [file EAT-59-546-s001.docx]

**Online Supplementary Material**

| **Table S1.**  *Prediction of eating disorder symptom severity. Subscale “eating concern”: Emotions and Word Count. Relative SHAP Value. and Correlations with SHAP Value.* | | | | | | | | | | |
| --- | --- | --- | --- | --- | --- | --- | --- | --- | --- | --- |
|  | **Eating Disorder Examination-Questionnaire: Subscale Eating Concern** | | | | | | | | | |
| Emotions | **Without ED diagnosis as a predictor** | | | | |  | **With ED diagnosis as a predictor** | | | |
|  | **RSV** | ***r*** | | ***p*** | ***p_adj_*** |  | **RSV** | ***r*** | ***p*** | ***p_adj_*** |
|  |  |  | |  |  | diagnosis | 85.62 % | > -.99 | < .001*** | < .001*** |
| joy | 11.45 % | -.82 | | < .001*** | < .001*** | disgust | 4.80 % | .87 | < .001*** | < .001*** |
| grief | 10.73 % | .75 | | < .001*** | < .001*** | caring | 1.40 % | .11 | .098 | .434 |
| disgust | 9.09 % | .75 | | < .001*** | < .001*** | joy | 1.18 % | -.60 | < .001*** | < .001*** |
| approval | 8.82 % | -.68 | | < .001*** | < .001*** | relief | 0.92 % | -.15 | .097 | .434 |
| remorse | 8.72 % | .86 | | < .001*** | < .001*** | sadness | 0.85 % | .05 | .776 | .962 |
| sadness | 8.40 % | .76 | | < .001*** | < .001*** | remorse | 0.57 % | .45 | .006* | .050 |
| relief | 7.92 % | -.53 | | < .001*** | < .001*** | annoyance | 0.56 % | .10 | .293 | .489 |
| fear | 6.03 % | .63 | | < .001*** | < .001*** | embarrassment | 0.46 % | .18 | .187 | .488 |
| optimism | 5.50 % | -.63 | | < .001*** | < .001*** | confusion | 0.43 % | -.14 | .130 | .488 |
| nervousness | 4.74 % | .48 | | < .001*** | < .001*** | pride | 0.42 % | -.01 | .841 | .965 |
| disapproval | 3.29 % | .63 | | < .001*** | < .001*** | neutral | 0.41 % | -.06 | .660 | .915 |
| neutral | 3.00 % | -.13 | | .537 | .701 | anger | 0.29 % | -.08 | .300 | .489 |
| realization | 2.43 % | -.20 | | .257 | .430 | grief | 0.28 % | .15 | .266 | .489 |
| surprise | 2.30 % | .14 | | .583 | .729 | approval | 0.27 % | -.15 | .359 | .556 |
| pride | 2.15 % | -.32 | | .055 | .117 | optimism | 0.21 % | -.02 | .833 | .965 |
| words | 1.36 % | -.23 | | .046* | .106 | words | 0.18 % | -.05 | .251 | .489 |
| love | 0.98 % | -.26 | | .026* | .064 | disapproval | 0.18 % | -.02 | .671 | .915 |
| confusion | 0.90 % | -.19 | | .120 | .225 | nervousness | 0.17 % | -.10 | .200 | .488 |
| embarrassment | 0.89 % | .10 | | .263 | .430 | surprise | 0.12 % | .08 | .268 | .489 |
| excitement | 0.49 % | -.10 | | .300 | .430 | gratitude | 0.12 % | .07 | .205 | .488 |
| anger | 0.47 % | .20 | | .103 | .207 | love | 0.09 % | .01 | .933 | > .99 |
| desire | 0.18 % | -.07 | | .301 | .430 | realization | 0.07 % | .02 | .714 | .923 |
| amusement | 0.17 % | .09 | | .278 | .430 | fear | 0.06 % | -.16 | .142 | .488 |
| admiration | 0.00 % | .00 | | .319 | .434 | excitement | 0.04 % | .02 | .679 | .915 |
| annoyance | 0.00 % | .00 | | > .99 | > .99 | desire | 0.00 % | -.03 | .066 | .407 |
| caring | 0.00 % | .00 | | > .99 | > .99 | disappointment | 0.00 % | .01 | .300 | .489 |
| curiosity | 0.00 % | .00 | | > .99 | > .99 | admiration | 0.00 % | .00 | > .99 | > .99 |
| disappointment | 0.00 % | .00 | | > .99 | > .99 | amusement | 0.00 % | .00 | > .99 | > .99 |
| gratitude | 0.00 % | .00 | | > .99 | > .99 | curiosity | 0.00 % | .00 | > .99 | > .99 |
| **Note.** ED = eating disorder; RSV = Relative SHAP Value i.e., percentage contribution of emotion to the prediction of self-reported symptom severity; *r* = Correlation with SHAP Value. significance levels of *p*-values * < .05, ** < .005, *** < .001; *p_adj._* = Benjamini-Hochberg adjustment for multiple testing of *p*-value | | | | | | | | | | |
|  |  | |  | | | | | |  |  |

| **Table S2.**  *Prediction of eating disorder symptom severity. Subscale “restraint”: Emotions and Word Count. Relative SHAP Value. and Correlations with SHAP Value.* | | | | | | | | | | |
| --- | --- | --- | --- | --- | --- | --- | --- | --- | --- | --- |
|  | **Eating Disorder Examination-Questionnaire: Subscale Restraint** | | | | | | | | | |
| Emotions | **Without ED diagnosis as a predictor** | | | | |  | **With ED diagnosis as a predictor** | | | |
|  | **RSV** | ***r*** | | ***p*** | ***p_adj_*** |  | **RSV** | ***r*** | ***p*** | ***p_adj_*** |
|  |  |  | |  |  | diagnosis | 59.54 % | -.99 | < .001*** | < .001*** |
| disgust | 13.94 % | .78 | | < .001*** | < .001*** | sadness | 7.42 % | -.78 | < .001*** | < .001*** |
| approval | 11.15 % | -.70 | | < .001*** | < .001*** | approval | 4.65 % | -.51 | < .001*** | .001** |
| grief | 10.95 % | .80 | | < .001*** | < .001*** | grief | 3.65 % | .58 | .001** | .006* |
| nervousness | 9.74 % | .84 | | < .001*** | < .001*** | nervousness | 2.95 % | .42 | .002** | .010* |
| sadness | 6.67 % | .37 | | .025* | .069 | neutral | 2.53 % | -.20 | .069 | .237 |
| relief | 5.04 % | -.36 | | .006* | .019* | gratitude | 2.49 % | .39 | .006* | .029 |
| neutral | 4.90 % | .10 | | .627 | .697 | relief | 2.44 % | -.06 | .707 | > .99 |
| optimism | 4.55 % | -.28 | | .005* | .017* | disgust | 1.99 % | .14 | .129 | .382 |
| pride | 4.35 % | -.61 | | < .001*** | < .001*** | surprise | 1.84 % | -.03 | .898 | > .99 |
| surprise | 3.86 % | .16 | | .333 | .416 | realization | 1.82 % | .18 | .187 | .478 |
| remorse | 3.86 % | .45 | | < .001*** | .001** | love | 1.64 % | -.13 | .242 | .478 |
| joy | 3.38 % | -.33 | | .004** | .017* | caring | 1.59 % | -.16 | .243 | .478 |
| realization | 2.85 % | -.13 | | .367 | .441 | remorse | 1.24 % | .11 | .407 | .701 |
| fear | 2.78 % | .14 | | .069 | .160 | excitement | 0.73 % | -.03 | .803 | > .99 |
| disapproval | 2.46 % | 0,35 | | .005* | .017* | joy | 0.57 % | -.15 | .136 | .382 |
| anger | 1.72 % | 0,16 | | .048* | .120 | pride | 0.35 % | -.24 | .060 | .232 |
| love | 1.12 % | -0,08 | | .262 | .402 | disappointment | 0.25 % | .15 | .205 | .478 |
| caring | 0.98 % | -0,08 | | .286 | .402 | fear | 0.16 % | -.10 | .255 | .478 |
| words | 0.56 % | -0,09 | | .149 | .297 | optimism | 0.00 % | -.02 | .262 | .478 |
| excitement | 0.55 % | 0,07 | | .437 | .504 | admiration | 0.00 % | .00 | > .99 | > .99 |
| embarrassment | 0.55 % | 0,06 | | .307 | .402 | amusement | 0.00 % | .00 | > .99 | > .99 |
| desire | 0.49 % | -0,10 | | .308 | .402 | anger | 0.00 % | .00 | > .99 | > .99 |
| curiosity | 0.36 % | -0,08 | | .305 | .402 | annoyance | 0.00 % | .00 | > .99 | > .99 |
| amusement | 0.32 % | 0,07 | | .276 | .402 | confusion | 0.00 % | .00 | > .99 | > .99 |
| disappointment | 0.21 % | 0,03 | | .277 | .402 | curiosity | 0.00 % | .00 | > .99 | > .99 |
| gratitude | 0.00 % | -0,02 | | .284 | .402 | desire | 0.00 % | .00 | > .99 | > .99 |
| admiration | 0.00 % | 0,00 | | > .99 | > .99 | disapproval | 0.00 % | .00 | > .99 | > .99 |
| annoyance | 0.00 % | 0,00 | | > .99 | > .99 | embarrassment | 0.00 % | .00 | > .99 | > .99 |
| confusion | 0.00 % | 0,00 | | > .99 | > .99 | words | 0.00 % | .00 | > .99 | > .99 |
| **Note.** ED = eating disorder; RSV = Relative SHAP Value i.e., percentage contribution of emotion to the prediction of self-reported symptom severity; *r* = Correlation with SHAP Value. significance levels of *p*-values * < .05, ** < .005, *** < .001; *p_adj._* = Benjamini-Hochberg adjustment for multiple testing of *p*-value | | | | | | | | | | |
|  |  | |  | | | | | |  |  |

| **Table S3.**  *Prediction of eating disorder symptom severity. Subscale “shape concern”: Emotions and Word Count. Relative SHAP Value. and Correlations with SHAP Value.* | | | | | | | | | | |
| --- | --- | --- | --- | --- | --- | --- | --- | --- | --- | --- |
|  | **Eating Disorder Examination-Questionnaire: Subscale Shape Concern** | | | | | | | | | |
| Emotions | **Without ED diagnosis as a predictor** | | | | |  | **With ED diagnosis as a predictor** | | | |
|  | **RSV** | ***r*** | | ***p*** | ***p_adj_*** |  | **RSV** | ***r*** | ***p*** | ***p_adj_*** |
|  |  |  | |  |  | diagnosis | 77.67 % | > -.99 | < .001*** | < .001*** |
| joy | 24.26 % | -.70 | | < .001*** | < .001*** | optimism | 4.14 % | -.51 | < .001*** | < .001*** |
| optimism | 10.88 % | -.77 | | < .001*** | < .001*** | joy | 3.20 % | -.62 | < .001*** | < .001*** |
| disgust | 9.81 % | .75 | | < .001*** | < .001*** | sadness | 1.91 % | -.45 | < .001*** | .001** |
| grief | 8.10 % | .54 | | < .001*** | < .001*** | disgust | 1.90 % | .46 | .003** | .011* |
| sadness | 7.21 % | .61 | | < .001*** | < .001*** | grief | 1.17 % | .49 | < .001*** | .002** |
| approval | 4.86 % | -.63 | | < .001*** | < .001*** | approval | 1.15 % | -.27 | .009* | .037* |
| fear | 4.37 % | .43 | | < .001*** | < .001*** | caring | 1.02 % | .23 | .092 | .204 |
| relief | 4.36 % | -.15 | | .172 | .283 | anger | 0.75 % | .23 | .056 | .145 |
| nervousness | 4.05 % | .37 | | .002** | .005* | relief | 0.74 % | -.14 | .129 | .267 |
| remorse | 4.00 % | .67 | | < .001*** | < .001*** | realization | 0.71 % | .00 | .942 | > .99 |
| surprise | 3.16 % | .65 | | < .001*** | < .001*** | disapproval | 0.70 % | .39 | .001** | .007* |
| realization | 3.07 % | .12 | | .261 | .354 | surprise | 0.63 % | .14 | .370 | .521 |
| confusion | 2.01 % | -.37 | | .001** | .004* | remorse | 0.61 % | .10 | .406 | .524 |
| pride | 1.96 % | -.30 | | .020* | .045* | pride | 0.59 % | -.19 | .027* | .083 |
| admiration | 1.45 % | -.18 | | .179 | .283 | words | 0.59 % | -.04 | .733 | .874 |
| disappointment | 1.39 % | .22 | | .109 | .217 | embarrassment | 0.57 % | .00 | .998 | > .99 |
| disapproval | 1.34 % | .39 | | .002* | .005* | disappointment | 0.41 % | -.09 | .041* | .114 |
| neutral | 1.02 % | .17 | | .269 | .354 | neutral | 0.38 % | -.09 | .570 | .707 |
| desire | 0.66 % | -.24 | | .042* | .090 | excitement | 0.36 % | -.03 | .399 | .524 |
| anger | 0.43 % | .09 | | .283 | .354 | fear | 0.19 % | -.11 | .021* | .074 |
| love | 0.37 % | -.17 | | .142 | .250 | confusion | 0.19 % | -.08 | .152 | .294 |
| words | 0.35 % | -.08 | | .295 | .354 | love | 0.14 % | -.04 | .295 | .441 |
| excitement | 0.26 % | .06 | | .116 | .217 | nervousness | 0.08 % | -.01 | .299 | .441 |
| embarrassment | 0.25 % | .08 | | .312 | .360 | desire | 0.06 % | -.03 | .091 | .204 |
| annoyance | 0.23 % | .08 | | .281 | .354 | curiosity | 0.02 % | .06 | .285 | .441 |
| amusement | 0.00 % | .00 | | > .99 | > .99 | admiration | 0.00 % | -.03 | .290 | .441 |
| caring | 0.00 % | .00 | | > .99 | > .99 | amusement | 0.00 % | .00 | > .99 | > .99 |
| curiosity | 0.00 % | .00 | | > .99 | > .99 | annoyance | 0.00 % | .00 | > .99 | > .99 |
| gratitude | 0.00 % | .00 | | > .99 | > .99 | gratitude | 0.00 % | .00 | > .99 | > .99 |
| **Note.** ED = eating disorder; RSV = Relative SHAP Value i.e., percentage contribution of emotion to the prediction of self-reported symptom severity; *r* = Correlation with SHAP Value. significance levels of *p*-values * < .05, ** < .005, *** < .001; *p_adj._* = Benjamini-Hochberg adjustment for multiple testing of *p*-value | | | | | | | | | | |
|  |  | |  | | | | | |  |  |

| **Table S4.**  *Prediction of eating disorder symptom severity. Subscale “weight concern”: Emotions and Word Count. Relative SHAP Value. and Correlations with SHAP Value.* | | | | | | | | | | |
| --- | --- | --- | --- | --- | --- | --- | --- | --- | --- | --- |
|  | **Eating Disorder Examination-Questionnaire: Subscale Weight Concern** | | | | | | | | | |
| Emotions | **Without ED diagnosis as a predictor** | | | | |  | **With ED diagnosis as a predictor** | | | |
|  | **RSV** | ***r*** | | ***p*** | ***p_adj_*** |  | **RSV** | ***r*** | ***p*** | ***p_adj_*** |
|  |  |  | |  |  | diagnosis | 92.10 % | > -.99 | < .001*** | < .001*** |
| joy | 15.41 % | -.79 | | < .001*** | < .001*** | disgust | 2.51 % | .59 | < .001*** | .001** |
| optimism | 11.82 % | -.76 | | < .001*** | < .001*** | sadness | 1.38 % | -.11 | .166 | .682 |
| disgust | 11.65 % | .84 | | < .001*** | < .001*** | disappointment | 0.71 % | -.08 | .206 | .682 |
| approval | 8.19 % | -.60 | | < .001*** | < .001*** | joy | 0.49 % | -.10 | .173 | .682 |
| sadness | 6.39 % | .43 | | .014* | .034* | words | 0.47 % | .02 | .601 | .980 |
| relief | 6.25 % | -.24 | | .055 | .103 | surprise | 0.43 % | .09 | .400 | .781 |
| grief | 5.81 % | .41 | | .004** | .013* | caring | 0.34 % | .06 | .454 | .781 |
| remorse | 5.75 % | .58 | | < .001*** | .001** | relief | 0.34 % | -.09 | .197 | .682 |
| surprise | 4.05 % | .36 | | .029* | .067 | optimism | 0.33 % | -.10 | .308 | .682 |
| confusion | 4.02 % | -.46 | | < .001*** | .001** | realization | 0.25 % | -.01 | .915 | > .99 |
| realization | 3.49 % | .40 | | .013* | .034* | approval | 0.24 % | -.03 | .707 | .996 |
| embarrassment | 2.56 % | .42 | | .002** | .007* | grief | 0.12 % | .13 | .217 | .682 |
| fear | 2.28 % | .11 | | .181 | .259 | disapproval | 0.10 % | .08 | .170 | .682 |
| neutral | 2.22 % | -.26 | | .084 | .149 | anger | 0.08 % | .07 | .403 | .781 |
| words | 2.15 % | -.24 | | .050 | .100 | neutral | 0.07 % | .04 | .632 | .980 |
| love | 2.00 % | -.29 | | .042* | .089 | remorse | 0.03 % | -.02 | .672 | .992 |
| nervousness | 1.92 % | .14 | | .125 | .188 | fear | 0.00 % | -.02 | .453 | .781 |
| disapproval | 1.20 % | .33 | | .001** | .005* | gratitude | 0.00 % | .03 | .206 | .682 |
| desire | 1.06 % | -.31 | | .008* | .023 | amusement | 0.00 % | -.01 | .283 | .682 |
| anger | 0.85 % | .15 | | .120 | .188 | embarrassment | 0.00 % | .02 | .884 | > .99 |
| pride | 0.56 % | -.18 | | .117 | .188 | desire | 0.00 % | .07 | .304 | .682 |
| admiration | 0.26 % | -.05 | | .300 | .381 | admiration | 0.00 % | .02 | .292 | .682 |
| gratitude | 0.00 % | .02 | | .292 | .381 | pride | 0.00 % | .01 | .305 | .682 |
| amusement | 0.00 % | .00 | | > .99 | > .99 | annoyance | 0.00 % | .00 | > .99 | > .99 |
| annoyance | 0.00 % | .00 | | > .99 | > .99 | confusion | 0.00 % | .00 | > .99 | > .99 |
| caring | 0.00 % | .00 | | > .99 | > .99 | curiosity | 0.00 % | .00 | > .99 | > .99 |
| curiosity | 0.00 % | .00 | | > .99 | > .99 | excitement | 0.00 % | .00 | > .99 | > .99 |
| disappointment | 0.00 % | .00 | | > .99 | > .99 | love | 0.00 % | .00 | > .99 | > .99 |
| excitement | 0.00 % | .00 | | > .99 | > .99 | nervousness | 0.00 % | .00 | > .99 | > .99 |
| **Note.** ED = eating disorder; RSV = Relative SHAP Value i.e., percentage contribution of emotion to the prediction of self-reported symptom severity; *r* = Correlation with SHAP Value. significance levels of *p*-values * < .05, ** < .005, *** < .001; *p_adj._* = Benjamini-Hochberg adjustment for multiple testing of *p*-value | | | | | | | | | | |
|  |  | |  | | | | | |  |  |

| ****Table S5.** *Hyperparameters without including diagnosis as a predictor.*** | |
| --- | --- |
| **Test fold** | **Hyperparameter list** |
| 1. **SVR** | **{'model__C': 2. 'model__coef0': 1e-20. 'model__degree': 2. 'model__gamma': 'auto'. 'model__kernel': 'rbf'}** |
| 1. **RF** | **{'bootstrap': True. 'max_depth': 10. 'max_features': 20. 'min_samples_leaf': 2. 'min_samples_split': 2. 'n_estimators': 100}** |
| 1. **RF** | **{'bootstrap': True. 'max_depth': 10. 'max_features': 10. 'min_samples_leaf': 2. 'min_samples_split': 2. 'n_estimators': 100}** |
| 1. **RF** | **{'bootstrap': True. 'max_depth': 10. 'max_features': 10. 'min_samples_leaf': 2. 'min_samples_split': 2. 'n_estimators': 100}** |
| 1. **RF** | **{'bootstrap': True. 'max_depth': 5. 'max_features': 10. 'min_samples_leaf': 2. 'min_samples_split': 2. 'n_estimators': 100}** |
| 1. **RF** | **{'bootstrap': True. 'max_depth': 5. 'max_features': 10. 'min_samples_leaf': 2. 'min_samples_split': 2. 'n_estimators': 100}** |
| 1. **RF** | **{'bootstrap': True. 'max_depth': 5. 'max_features': 10. 'min_samples_leaf': 2. 'min_samples_split': 2. 'n_estimators': 100}** |
| 1. **RF** | **{'bootstrap': True. 'max_depth': 15. 'max_features': 10. 'min_samples_leaf': 4. 'min_samples_split': 2. 'n_estimators': 100}** |
| 1. **RF** | **{'model__C': 6. 'model__coef0': 1e-20. 'model__degree': 2. 'model__gamma': 'auto'. 'model__kernel': 'rbf'}** |
| 1. **RF** | **{'model__C': 1. 'model__coef0': 1e-20. 'model__degree': 2. 'model__gamma': 'auto'. 'model__kernel': 'rbf'}** |

| ****Table S6.** *Hyperparameters with diagnosis as a predictor*** | |
| --- | --- |
| **Test fold** | **Hyperparameter list** |
| 1. **Lasso** | **{'model__alpha': 0.165}** |
| 1. **RF** | **{'bootstrap': True. 'max_depth': 15. 'max_features': 30. 'min_samples_leaf': 4. 'min_samples_split': 2. 'n_estimators': 100}** |
| 1. **RF** | **{'bootstrap': True. 'max_depth': 15. 'max_features': 30. 'min_samples_leaf': 4. 'min_samples_split': 2. 'n_estimators': 1000}** |
| 1. **RF** | **{'model__alpha': 0.19500000000000003}** |
| 1. **Lasso** | **{'model__alpha': 0.17500000000000002}** |
| 1. **Lasso** | **{'model__alpha': 0.15000000000000002}** |
| 1. **Lasso** | **{'model__alpha': 0.22000000000000003}** |
| 1. **RF** | **{'bootstrap': True. 'max_depth': 15. 'max_features': 10. 'min_samples_leaf': 4. 'min_samples_split': 2. 'n_estimators': 100}** |
| 1. **Lasso** | **{'model__alpha': 0.21500000000000002}** |
| 1. **RF** | **{'bootstrap': True. 'max_depth': 15. 'max_features': 30. 'min_samples_leaf': 4. 'min_samples_split': 2. 'n_estimators': 100}** |

| **Table S7.** *Feature Importance Correlation Separated for RF and Lasso* | | |
| --- | --- | --- |
| **Feature** | **Percent Importance Lasso** | **Percent Importance RF** |
| words | 5.10847E-14 | 1.506362794 |
| admiration | 0 | 0 |
| amusement | 0 | 0 |
| anger | 6.13119E-14 | 1.476422433 |
| annoyance | 0 | 0 |
| approval | 9.05752E-13 | 1.305827769 |
| caring | 2.04361E-14 | 4.441066441 |
| confusion | 1.05119E-12 | 0 |
| curiosity | 0 | 0 |
| desire | 0 | 0.086368457 |
| disappointment | 9.27346E-13 | 0.715901392 |
| disapproval | 3.06596E-14 | 0.322890735 |
| disgust | 2.814603246 | 3.331120558 |
| embarrassment | 1.06945E-12 | 0.945611885 |
| excitement | 5.40947E-13 | 0 |
| fear | 8.12529E-13 | 0.582929693 |
| gratitude | 0 | 0 |
| grief | 0 | 0.141294029 |
| joy | 1.61348E-13 | 1.307521819 |
| love | 3.74468E-13 | 0 |
| nervousness | 0 | 0.126580068 |
| optimism | 4.31282E-13 | 4.479738407 |
| pride | 1.66038E-12 | 1.097653023 |
| realization | 3.62534E-13 | 1.024286493 |
| relief | 1.03076E-12 | 1.97566523 |
| remorse | 1.25297E-12 | 0.652166697 |
| sadness | 7.65922E-13 | 3.251993716 |
| surprise | 9.4666E-13 | 1.485682452 |
| neutral | 1.25808E-12 | 0.867297755 |
| Diagnosis | 97.18539675 | 68.87561815 |
